# Efficacy of Acupuncture for Psychological Symptoms Associated with Opioid Addiction: A Systematic Review and Meta-Analysis

**DOI:** 10.1155/2014/313549

**Published:** 2014-11-04

**Authors:** Zhang Boyuan, Chen Yang, Cheng Ke, Shen Xueyong, Liu Sheng

**Affiliations:** ^1^Shanghai University of Traditional Chinese Medicine, 1200 Cailun Road, Shanghai 200032, China; ^2^College of Traditional Chinese Medicine, Xinjiang Medical University, Ürümqi 830011, China

## Abstract

This review systematically assessed the clinical evidence for and against acupuncture as a treatment for psychological symptoms associated with opioid addiction. The database was accessed from MEDLINE and China Knowledge Resource Integrated Database. We included all randomized clinical trials published in Chinese and English regardless of their controls. Meta-analysis was performed using the RevMan software, version 5.2. We conducted a literature search of 16 databases from their inception to January 2014. Four studies from Western countries did not report any clinical gains in the treatment of psychological symptoms associated with opioid addiction. 10 of 12 studies from China have reported positive findings regarding the use of acupuncture to treat the psychological symptoms associated with opioid addiction. The methodological quality of the included studies was poor. The meta-analysis indicated that there was a significant difference between the treatment group and the control group for anxiety and depression associated with opioid addiction, although groups did not differ on opioid craving. This review and meta-analysis could not confirm that acupuncture was an effective treatment for psychological symptoms associated with opioid addiction. However, considering the potential of acupuncture demonstrated in the included studies, further rigorous randomized controlled trials with long followup are warranted.

## 1. Introduction

Opioid dependence, most commonly manifested as heroin dependence, remains a significant public health problem around the world. Clinically, opioid dependence is characterized by physical dependence, as evidenced by tolerance and withdrawal, and by psychological symptoms, including drug cravings, depression, anxiety, and inability to control heroin use. Conventional detoxification methods such as methadone and buprenorphine are effective in reducing illicit opioid use, but problems associated with their use, such as social resistance to the idea of “replacing one drug of abuse with another” and difficulties in tapering patients off the medication due to long-lasting withdrawal effects, make the search for alternative therapies important [1].

Opioid detoxification addresses the first steps in the treatment of heroin dependence. Most detoxification methods are designed to decrease withdrawal-related discomfort and complications but do not address the psychological symptoms associated with opioid addiction [2, 3]. The epidemiological studies have revealed important facts about psychiatric comorbidity in opioid addicts [4, 5]. Psychiatric comorbidity between anxiety, depressive, and substance use disorders is very common, with a third to a half of persons with any mental disorder meeting criteria for another mental or substance use disorder at some point in their lives [5]. Heroin addicts with comorbid mental disorders often have a poorer treatment response and a worse course of illness over time [5, 6]. In addition, accumulating evidence suggests that psychological symptoms and other emotional factors contribute the most to heroin relapse [7, 8]. Heiwe et al. [9] showed that the presence of depressive symptoms when initiating the withdrawal process was a risk factor for both dropping out from the withdrawal process and relapse after completion of the withdrawal process. Common psychological symptoms such as anxiety and depression are often associated with initiation and use of opioid [10].

Acupuncture originated in ancient China and has been used to manage various clinical disorders for thousands of years. A survey of acupuncture released by an NIH Consensus Development Panel indicated that acupuncture is beneficial in treating various pain syndromes, postoperative and chemotherapy-induced nausea and vomiting, some forms of bronchial asthma, headache, migraine, and female infertility [11–19]. Recently, acupuncture or electroacupuncture (EA) has been applied to attenuate behavioral signs of heroin withdrawal in addicts [20–24]. However, the findings in the clinical literature regarding the effectiveness of acupuncture in opioid dependence have been inconsistent. Also, the previous reviews and meta-analysis cannot be used to establish the efficacy of acupuncture in the treatment of opiate addiction [25–30]. These findings have challenged the field to consider the proper role of acupuncture treatment.

There are some variable factors that need to be taken into account when assessing the effects of acupuncture on opioid addiction. Firstly, the study protocol may influence the assessment of effectiveness of acupuncture. Methods and research designs have been issues of debate among acupuncture clinicians and researchers [31]. For a methodological perspective, randomized controlled trials (RCTs) are considered the gold standard in terms of identifying differences in treatment efficacy [32]. However, unlike the evaluation of a new drug, RCTs of acupuncture are extremely difficult to conduct, particularly if they have to be blind in design, and acupuncture has to be compared with a placebo [33]. Secondly, in China, approximately 1.16 million drug addicts were officially registered in late 2005, but unofficial estimates place this number closer to 3.5 million [34]. The predominantly abused drug is heroin, and the majority of addicts use this drug by injection. The literature describing and researching the effect of acupuncture on opioid addiction was mainly published in Chinese language journals, making it not accessible to the Western readers. While some publications on acupuncture in Chinese language journals may not meet the stringent requirements of international peer-reviewed journals, they may still provide potentially useful observations and ideas for further study. Thirdly, most previous reviews systematically analyzing acupuncture for the treatment of opioid addiction focused on controlling opioid withdrawal but not on psychological symptoms associated with opioid addiction [29, 30, 35, 36].

This paper provides an overview of the clinical studies that have investigated the clinical effectiveness of acupuncture and focused on psychological symptoms associated with opioid addiction. The clinical studies published in Chinese language journals were assessed carefully and included in our systematical reviews. We also summarize the quality of the study design, types of acupuncture applied, commonly selected acupoints or sites of the body, and effectiveness of the treatment in these studies.

## 2. Methods

### 2.1. Data Source and Study Selection

The following sources were all searched from their inception to January 2014: MEDLINE (comprises more than 23 million citations for biomedical literature from MEDLINE, life science journals, and online books) and China Knowledge Resource Integrated Database (the largest database in China, including China Academic Journals Full-Text Database, China Doctoral/Master Dissertations Full-Text Database, China Proceedings Conference Full-Text Database, and China Year Books Full-Text Database). Personal contact was made with the authors of the published studies, if necessary, to request for additional data. We also extended our search spectrum to the “related articles” and bibliographies of all retrieved studies. If multiple publications from the same study group occurred, we included only the most complete article for analysis.

The search required at least one term from each of the following categories: (1) heroin, opiate, and opioid; (2) acupuncture and electroacupuncture; (3) withdrawal syndromes, retention, dropout, completion, positive urine, craving, abstinence, heroin use, depression, anxiety, and relapse. In addition, we focused on the effect of acupuncture on psychological symptoms associated with opioid addiction. Although some studies satisfied the conditions mentioned above, only studies that reported findings on opioid craving, depression, or anxiety were included.

Two reviewers (ZB, CY), working independently and in duplicate, assessed the abstracts of all the studies meeting the above-mentioned criteria to ensure that they met the following criteria: (1) full text was available in English and Chinese, (2) study was of human subjects, (3) randomized control trials adopted a double-blind, single-blind, or nonblind design, and (4) when the study population of two or more research articles included the same, or some of the same, participants, the article that described the largest population was used. Exclusion criteria included (1) the nonnumeric data, (2) the comments and replies, (3) the animal study, (4) the mechanism study, and (5) the fact that the publication was a review article.

The full manuscripts were retrieved for studies with abstracts that met these criteria. The studies were again reviewed by the two reviewers to ensure that all of the above criteria were met in the full text. Disagreements in the final selection were resolved by consensus, and in cases of continuing disagreement, through consultation with a third reviewer (LS). The reviewers also recorded and compared their reasons for excluding studies, and a consensus was reached when there were disagreements. Dr. Liu monitored the whole process of systematic review. All reviewers were fully trained in the systematic review process executed.

### 2.2. Data Extraction and Quality Assessment

Data were extracted from study reports by one reviewer (ZB) and were verified by the second reviewer (CY). The following key information was extracted from each study: first author, publication year, study design, sample size, characteristics of participants, main acupoints/sites selected, outcome measures, and results reported (opioid/heroin carving, anxiety, and depression). We assessed the quality of the studies using the Jadad scale [33], which rates studies for (1) random sequence generation, (2) randomization concealment, (3) description of blinding, and (4) description of withdrawal. Studies scoring 1 point or 2 points were considered of low quality whereas studies scoring 3–5 points were considered of high quality. The Jadad scale is a procedure to independently assess the methodological quality of a clinical trial. It is the most widely used assessment in the world [37]. Lin et al. reported on the use of the Jadad scale in assessing efficacy of acupuncture for opiate dependence [28]. Given that some publications on acupuncture in Chinese language journals may not meet the stringent requirements of international peer-reviewed journals, only the clinical trials scoring ≥1 points were included for further assessment.

The clinical studies selected were divided into four categories: (1) acupuncture is compared to standard treatment; (2) acupuncture is compared to placebo treatment; (3) acupuncture at particular acupoints along particular classical meridians is compared to acupuncture at putatively ineffective acupoints/sites; and (4) acupuncture is compared with an untreated group.

### 2.3. Data Synthesis and Meta-Analysis

Standard meta-analysis methods were used in the present study. We pooled all continuous data from trials according to their control interventions (sham acupuncture, no treatment, or drug) and outcome measures (craving, anxiety, and depression) used. Summary test statistics were calculated using the RevMan software, version 5.2 [38]. Random effects model was used to account for the expected heterogeneity. The heterogeneity was evaluated using the *I* ^2^ statistic, which indicates the proportion of variability across trials not explained by chance alone [39]. Forest plots were used to present pooled effect size and individual study effect sizes.

## 3. Results

### 3.1. Literature Review

**Figure 1:**
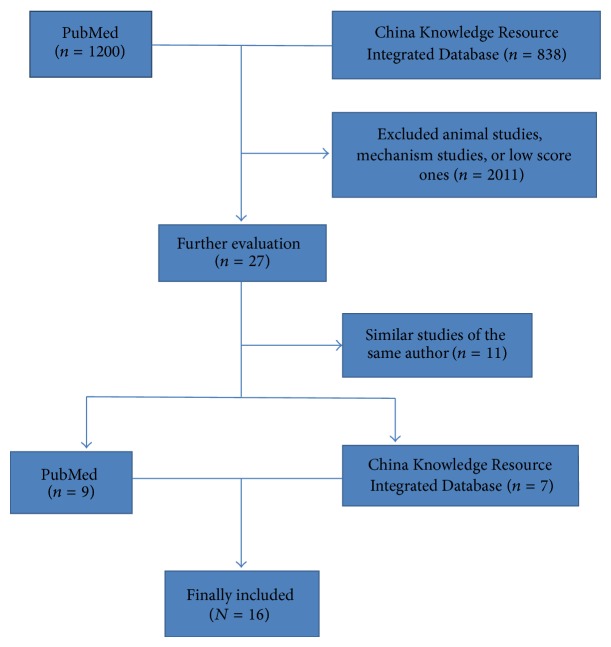
Flow diagram showing the number of studies included and excluded from the systematic review.

Figure 1 summarizes the findings of the literature review. Preliminary literature search criteria identified 1200 studies from PubMed and 838 studies from China Knowledge Resource Integrated Database. Further refinement and systematic review of full-text versions yielded 16 relevant studies (nine studies from PubMed and seven studies from China Knowledge Resource Integrated Database). The others were excluded for they were animal studies, mechanism studies, or similar studies by the same author.

Table 1 reports key characteristics of the included studies. For each study, the relevant sample size, inclusion criteria, intervention type, type of control group, follow-up periods, outcome measure, study duration, and Jadad scores are listed.  Table 2 provides details of acupuncture interventions in the included studies based on the recommendations on the standards for reporting interventions in controlled trials of acupuncture (STRICTA), including details of needling, treatment regimen, and practitioner background.

### 3.2. Types of Studies

Although all trials were randomized, only 11 trials described the randomization technique [40–43, 45, 47, 49–51, 53, 54]. One study [40] mentioned the process of randomization. Five studies [41–43, 49, 51] mentioned the use of blinding of clinicians, subjects, or raters of study outcomes. Allocation concealment was not adequately reported in all studies. Four studies were published in English language journals and 12 studies were published in Chinese language journals.

### 3.3. Diagnostic Criteria and Characteristics of Participants

Thirteen studies used the “*Diagnostic and Statistical Manual of Mental Disorders*” (DSM III, III-R, and IV) criteria on opiate or heroin dependence. One study [50] used the Chinese Classification of Mental Disorders (CCMD II-R). Two studies [40, 43] did not mention the criteria used in diagnosing opiate dependence. These studies involved 1599 subjects (including those in intervention groups and in control groups).

### 3.4. Type of Intervention and Needling Method

Four studies [40–43], all from English language journals, used auricular acupuncture. Seven studies [44–46, 49–51, 54] used body acupuncture with manual stimulation. Two studies [52, 55] used scalp acupuncture. Two studies [43, 48] used Han's acupoint nerve stimulator (HANS) for the treatment group.

For study categories, eight studies [40, 44, 46, 49–52, 54, 55] mentioned acupuncture compared to standard treatment. Three studies [41, 47, 48] mentioned acupuncture compared to placebo treatment. Two studies [42, 43] mentioned acupuncture at particular acupoints along particular classical meridians compared to acupuncture at putatively ineffective acupoints/sites. Four studies [45, 47, 50, 53] mentioned acupuncture compared with an untreated group.

### 3.5. Outcome Measures and Effectiveness Assessment

Eight studies [40–44, 47, 48, 52] included heroin/opioid craving. Seven studies [45, 46, 49, 51, 53–55] included anxiety. Two studies included depression [50, 53]. All of the four studies [40–43] published in English language journals did not show favorable effects of acupuncture on psychological symptoms associated with opioid addiction (anxiety, depression, and craving). 11 of 12 studies published in Chinese language journals supported use of acupuncture for controlling psychological symptoms associated with opioid addiction: craving [44, 47, 48, 52], anxiety [46, 48, 49, 51, 53, 55], and depression [50, 53]. The duration of the interventions was shorter than one month in 10 studies. No studies provided follow-up observation.

### 3.6. Main Acupoints/Sites Selected

Table 3 provides summary of main acupoints/sites selected in the reviewed studies. 15 studies used a fixed set of acupoints/sites on their subjects. Only one study [44] allowed some flexibility and needled additional points based on the symptom presentation of individual subjects. The five ear acupoints (sympathetic, Shenmen, kidney, lung, and liver) were used in four studies published in the English language journal. The most frequently used acupoints are Neiguan (PC6, 11.11%), Zusanli (ST36, 9.26%), Sanyinjiao (SP6, 7.41%), and Shenmen (HT7, 6.48%). A summary of the main acupoints/sites selected in the included studies is presented in Table 3.

### 3.7. Methodological Quality

All methodological quality scores are presented in Table 1. Six studies [40–43, 52, 55] scored ≥3 points and were classified as having higher quality. The other 10 studies scored <3 points and were considered as having lower quality. The mean Jadad scores of the studies published in English language journals were 3.50 (SEM = 0.29). The mean Jadad scores of the studies published in Chinese language journals were 1.92 (SEM = 0.20). The mean Jadad scores of the studies for and against acupuncture as a treatment for psychological symptoms associated with opioid addiction were 1.91 (SEM = 0.21) and 3.00 (SEM = 0.21), respectively.

### 3.8. Meta-Analysis for Psychological Symptoms Associated with Opioid Addiction

**Figure 2:**
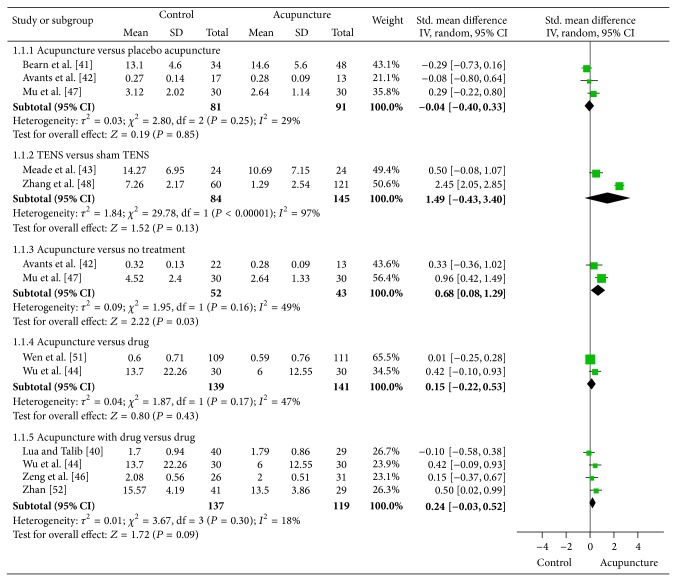
Meta-analysis of acupuncture for opioid craving.

Figure 2 reports the results of the meta-analysis of acupuncture for opioid craving. Nine trials [40–44, 46–48, 51] compared the effects of acupuncture with different control interventions for opioid craving. The pooled results indicated that there was no difference among acupuncture, placebo acupuncture (standard mean difference (SMD) = −0.04, 95% confidence interval: −0.04 to 0.33, and *I* ^2^ = 29%), sham transcutaneous electrical nerve stimulation (TENS) (SMD = 1.49, 95% CI: −0.43 to 3.40, and *I* ^2^ = 97%), and drug (SMD = 0.15, 95% CI: −0.22 to 0.53, and *I* ^2^ = 47%) on the craving scores. There was also no evidence of difference between acupuncture plus drug and drug alone (SMD = 0.24, 95% CI: −0.03 to 0.52, and *I* ^2^ = 18%). Acupuncture only showed a statistically significant benefit compared with no treatment (SMD = 0.68, 95% CI: 0.08 to 1.29, and *I* ^2^ = 49%). The heterogeneity was very high for the comparison of TENS versus sham TENS (i.e., *I* ^2^ = 97%). The reason for this considerable heterogeneity might be the different sham control used and the different treatment protocol used in the Zhang RCT [48] and the Meade RCT [43]. The sham control used in Zhang RCT [48] was placing electrodes on the same acupoints with no electrical stimulation, which might not be considered as a believable sham control. The sham control used in Meade RCT [43] was placing electrodes on the same acupoints with very weak electrical stimulation, which is more believable. The difference between the control and the treatment protocol may explain the larger effect size between the Zhang RCT and the Meade RCT.

**Figure 3:**
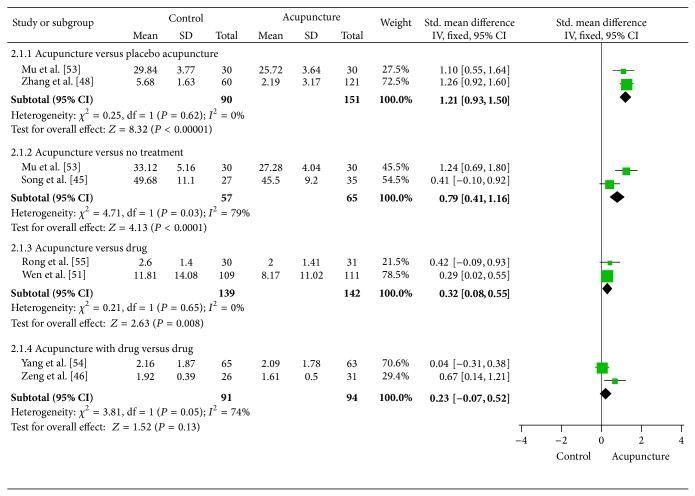
Meta-analysis of acupuncture for anxiety associated with opioid addiction.

The results of the meta-analysis for anxiety associated with opioid addiction are shown in Figure 3. Eight trials [45, 46, 48–50, 53–55] comparing acupuncture versus different control therapies found that acupuncture achieved a greater improvement than placebo acupuncture (SMD = 1.21, 95% CI: 0.93 to 1.50, and *I* ^2^ = 0%), drug (SMD = 0.32, 95% CI: 0.08 to 0.55, and *I* ^2^ = 0%), or no treatment therapies (SMD = 0.82, 95% CI: 0.00 to 1.64, and *I* ^2^ = 79%). Because both Yang RCT [49] and Mu RCT [53] used the same measurements in assessing anxiety (i.e., self-rating anxiety scale), we used the mean difference (MD) instead of SMD: the pooled effect was MD = 5.56 (95% CI: 3.42 to 7.69, and *I* ^2^ = 0%).

**Figure 4:**
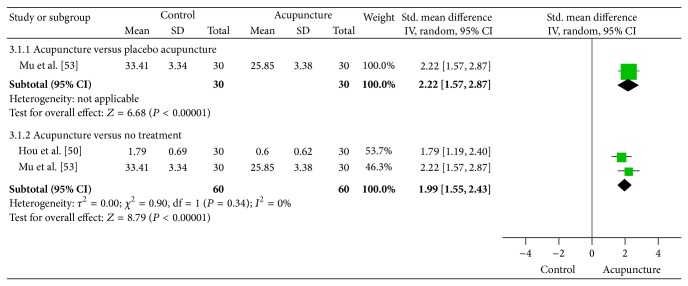
Meta-analysis of acupuncture for depression associated with opioid addiction.

Figure 4 reports the results of the meta-analysis of acupuncture for depression associated with opioid addiction. Two trials [50, 53] compared the effects of acupuncture with placebo acupuncture and no treatment. The results showed a statistically significant benefit of acupuncture for improving depression symptoms compared with placebo acupuncture (SMD = 2.22, 95% CI: 1.57 to 2.87, and *I* ^2^ = 0%) and compared with no treatment also (SMD = 1.99, 95% CI: 1.55 to 2.43, and *I* ^2^ = 0%).

## 4. Discussion

This is the first systematic review and meta-analysis on the effectiveness of acupuncture for treatment of psychological symptoms associated with opioid addiction. We identify and summarize the evidence about the possible clinical effectiveness of acupuncture on three psychological symptoms: heroin/opioid craving, depression, and anxiety. Unfortunately, the data does not allow us to make conclusions that acupuncture was an effective treatment for psychological symptoms associated with opioid addiction, given that most of studies reviewed here were hampered by small numbers of patients, lack of details regarding acupoints and needle manipulation, insufficient reporting of randomization and allocation concealment methods, improper blinding, and strength of the inference.

In our systematic review and meta-analysis, we distinguished the papers published in English language journals from those published in Chinese language journals. We found that most of the papers from China reported positive findings regarding the use of acupuncture to treat psychological symptoms associated with opioid addiction. However, all of four studies from Western countries did not report any clinical benefits from acupuncture for the treatment of psychological symptoms associated with opioid addiction. Although the discrepancy between these studies might be explained by the quality of research (studies published in English language journal received a higher Jadad quality scores), it has proved difficult to apply and integrate the basic principles and methodology of modern science that ensure the reliability of research subjects to clinical studies on acupuncture. The differences in patient selection, type of acupuncture treatments, acupoints selected, intensity, duration, and frequency of stimulation, total number of treatment sessions, and treatment period made the results hard to interpret. In fact, in traditional Chinese medical system, such as acupuncture, each individual is treated according to specific cases and symptoms. It may be unsound to use the same protocol for every case or condition. Individualized protocols are critical to the success of acupuncture treatment. After all, as a matter of clinical practice, the data reviewed here may be informative, as clinicians can attempt to ascertain which study design more closely resembles the nature of their practice. For example, most studies from China used body acupuncture to treat opiate addiction, whereas studies from the other countries used auricular acupuncture. These findings are intriguing considering that acupuncture on body and auricular points exhibited different efficacies. In addition, the most frequently used acupoints are Neiguan (PC6), Zusanli (ST36), Sanyinjiao (SP6), and Shenmen (HT7). According to our clinical experience and the theory of traditional Chinese medicine, these acupoints could effectively reduce anxiety and depression. Some acupoints, such as Shen Shu (BL.23) and Bai Hui (GV20), located on the back and head, can exhibit similar, if not identical, therapeutic effects. Further studies on the synergistic combination of acupoints could assist acupuncturists to use a balanced and appropriate choice for combining points in the treatment of psychological symptoms associated with opioid addiction.

Treatment retention and abstinence are more important goals for the treatment of opioid/heroin dependence. Effectiveness of treatment of psychological symptoms associated with opioid addiction should be assessed by including longer term follow-up data. In fact, to determine whether initial improvements from the treatment persist for a reasonable period of time, participant observation should last for at least three months. However, most of the studies reviewed in this meta-analysis did not provide follow-up data. In these studies, the duration of acupuncture interventions was also shorter than one month. In fact, it is unclear whether the extent to which acupuncture has therapeutic effects depends on the duration and frequency of acupuncture. Arguably, longer treatment periods are required for acupuncture to have any chance of showing clinical effects. These variable factors should be taken into account when assessing the effects of acupuncture. Future studies should therefore have sufficiently large samples, extended treatment, and follow-up periods.

The prevalence of depression and anxiety is very high in heroin and other drug addicts. Depression and anxiety after prolonged abstinence become main factors contributing to drug relapse and craving [8, 9]. Our meta-analysis indicated a statistically significant benefit of acupuncture for improving depression and anxiety associated with opioid addiction. Notably, most studies reviewed here focused on the effects of acupuncture on opioid/heroin physical detoxification. Psychological symptoms associated with opioid addiction are not their main outcome measures. This raises the possibility that the acupuncture protocols designed for psychological symptoms associated with opioid addiction may be more effective for the treatment of cravings, anxiety, and depression. Some evidence supported our hypothesis. The effect of acupuncture on depression (including depressive neurosis and depression following stroke) has been documented repeatedly in controlled studies [56, 57]. Acupuncture has been found to be more effective in depressive patients with decreased excretion of 3-methyl-4-hydroxy-phenylglycol [58, 59]. In addition, acupuncture has been used to improve psychological condition and lessen fatigue [56, 58, 59]. Therefore, it is very meaningful to pay close attention to the effects of acupuncture in the treatment of psychological symptoms associated with opioid addiction.

Some animal and clinical studies have provided important information for understanding the underlying mechanisms of acupuncture in the treatment of psychological symptoms associated with opioid addiction, although we still do not fully know how acupuncture works. In the treatment of drug craving and relapse to drug use, a nonendorphin mediated mechanism is probably involved. Yoon et al. [60] demonstrated that acupuncture suppressed ethanol-induced dopamine release in the rat nucleus accumbens through the GABA_*B*_ receptor. Chae et al. [61] showed that acupuncture treatment at ST.36 attenuated nicotine-induced locomotor activity through reduction of postsynaptic neuronal activity in the nucleus and striatum. These results suggest that acupuncture could play an important role in suppressing the reinforcing effects of ethanol and other drugs. Our recent study [62] showed that acupuncture attenuated elevated c-fos expression in the central nucleus of the amygdale (CeA) during morphine withdrawal in rats. Some studies emphasize that the motivational components of opiate withdrawal appear to be centrally mediated by limbic structures such as the nucleus accumbens and amygdala. Therefore, elevated c-fos expression in the CeA might be associated with the motivational components of opiate withdrawal. Our observation that acupuncture suppressed elevated c-fos expression in the CeA indicated that acupuncture might have some therapeutic benefit in the treatment of the negative motivational aspect of opiate addiction. In addition, the CeA and the basolateral amygdala have been extensively and differentially involved in associative learning and memory processes, attributing affective salience to environmental stimuli paired with drug effects [10]. One theory of the neural mechanisms of drug abuse focuses on various learning and memory systems in which the normal functions of these complex neural circuits become subverted leading to compulsive drug seeking behaviours [63]. In this model, drugs of abuse initiate plasticity mechanisms in different learning and memory systems that come to control behaviours of the individual over other preexisting memories. Experiences with addictive drugs are encoded and stored like other experiences except that drugs of abuse only mimic a subset of the action of natural reinforcers in the brain.

In conclusion, this review fills a gap in the literature, and thus, despite the limitations of our methodology, we believe that the benefits of illuminating this relevant topic overcome the limitations. Our systematic review and meta-analysis provides limited evidence for the effectiveness of acupuncture on psychological symptoms associated with opioid addiction. However, considering the potential of acupuncture demonstrated in the studies included here, further rigorous randomized controlled trials with long follow-up are warranted but need to overcome the many limitations of the current evidence.

## Figures and Tables

**Table 1:** Summary of studies included in the review.

Author (year)	Number of subjects (Acu/Control)	Inclusion criteria	Intervention type	Type of control group	Study duration	Follow-up periods	Outcome measure	Results reported	Jadad score	Database
Lua and Talib 2013 [40]	97 (55/42)	Dependence on opiates	AA	MMT	Two months	No	Craving	Better than AA *P* < 0.05	3	PubMed English Language Journal

Bearn et al. 2009 [41]	82 (41/41)	DSM-IV	AA	Placebo	Four weeks	No	MCS	NS	3	PubMed English Language Journal

Avants et al. 2000 [42]	82 (28/27/27)	DSM-IV-R	AA	Sham	Eight weeks	No	ASI SOCRATES version TCS	NS	4	PubMed English Language Journal

Meade et al. 2010 [43]	55 (26/29)	Diagnosis of opioid dependence	TEAS	Sham	Four days	No	Craving	NS	4	PubMed English Language Journal

Wu et al. 2003 [44]	120 (30/30/30/30)	CCMD-II-R DSM-III-R	Body acupuncture HAN's	Buprenorphine	Ten days	No	Craving	Acu with opioid more better *P* < 0.05	1	PubMed Chinese Language Journal

Song et al. 2012 [45]	62 (35/27)	DSM-III-R	Body acupuncture	No treatment	Eight weeks	No	Anxiety scores	NSD	2	PubMed Chinese Language Journal

Zeng et al. 2005 [46]	57 (31/26)	DSM-III-R	Body acupuncture	MMT	Ten days	No	Anxiety	Better than control *P* < 0.01	1	PubMed Chinese Language Journal

Mu et al. 2010 [47]	120 (30/30/30/30)	DSM-IV	Electro acupuncture	Placebo No treatment	Ten weeks	six months	Craving scores of VAS	Better than control *P* < 0.05	2	PubMed Chinese Language Journal

Zhang et al. 2000 [48]	181 (121/60)	DSM-III-R	HANS	Placebo	Two weeks	No	Craving Anxiety	Better than control *P* < 0.05	1	PubMed Chinese Language Journal

Yang et al. 2007 [49]	40 (20/20)	DSM-IV-TR	Body acupuncture AA	Body acupuncture	Four weeks	No	Anxiety	Better than acu control *P* < 0.01	2	CNKI Chinese Language Journal

Hou et al. 2009 [50]	60 (30/30)	ICD-10	Body acupuncture	No treatment	Three weeks	No	Depression	Better than control *P* < 0.01	2	CNKI Chinese Language Journal

Wen et al. 2005 [51]	220 (111/109)	DSW-IV	Body acupuncture	Drug	Ten days	No	Anxiety	Better than control *P* < 0.01	2	CNKI Chinese Language Journal

Zhan [52]	81 (34/47)	DSM-IV-TR	Scalp acupuncture	Methadone	One months	No	Craving score	Better than control *P* < 0.05	3	CNKI Chinese Language Journal

Mu et al. 2008 [53]	120 (30/30/30)	DSM-IV	electro acupuncture	No treatment	Ten weeks	No	Anxiety Depression	Both Acu group better than control *P* < 0.05 *P* < 0.05	2	CNKI Chinese Language Journal

Yang et al. 2011 [54]	128 (65/63)	DSM-III-R	Body acupuncture Drug	Drug	Twelve days	No	Anxiety	Same as the drug group *P* > 0.05	2	CNKI Chinese Language Journal

Rong et al. 2006 [55]	94 (33/31/30)	DSM-IV	Scalp and electro acupuncture	Body acupuncture Methadone	Ten days	No	Anxiety	Better than control *P* < 0.01	3	CNKI Chinese Language Journal

NS: not significant; AA: auricular acupuncture; HANS: Han's acupoint nerve stimulator; CCMD: Chinese Classification of Mental Disorders; DSM: The Diagnostic and Statistical Manual of Mental Disorders; VAS: visual analogue scale; ASI: Addiction severity Index; SOCRATES: the stage of Change Readiness and Treatment Eagerness Scale; TCS: Treatment Credibility Scale; MCS: the Maudsley Craving Scale; SOWS: Severity of Opiate Withdrawal Symptom Scale; NADA: National Acupuncture Detoxification Association; TEAS: transcutaneous electric acupoint stimulation; NSD: no statistical difference.

**Table 2:** Acupuncture interventions in the included studies based on the STRICTA recommendation.

Author	Details of needling					Treatment regimen	Practitioner background
	Insertion depth	Response sought	Stimulation method	Retention time	Needle type		
Lua and Talib 2013 [40]	1–3 mm	NR	NR	30 min	0.25 × 12.55 mm	3 times weekly for 2 months	Acupuncturist

Bearn et al. 2009 [41]	NR	NR	NR	30–40 min	0.25 × 30 mm	Once each weekday for 2 weeks	Qualified acupuncturists

Avants et al. 2000 [42]	1–3 mm	NR	NR	40 min	0.2 × 15 mm	Once each weekday for 8 weeks	Professional acupuncturists

Meade et al. 2010 [43]	NR	NR	TEAS	30 min	NR	Once daily for 4 days	NR

Wu et al. 2003 [44]	NR	De-qi response manual	Manipulated every 5 min	30 min	NR	1.3 times per day for 10 days	NR

Song et al. 2012 [45]	10–15 mm	De-qi response manual	Moxibustion Manipulated every 15 min	30 min	0.35 × 40 mm	Twice a week for 8 weeks	NR

Zeng et al. 2005 [46]	NR	NR	Manipulated every 10 min	30 min	NR	Once per day for 10 days	NR

Mu et al. 2010 [47]	15–30 min	De-qi response manual	Manipulated every 2 min and EA	20 min	0.32 × 50 mm	3 times per week for 10 weeks	NR

Zhang et al. 2000 [48]	—	Tolerance	HAN'S	NR	Electrode	2.1 times per day for 15 days	NR

Yang et al. 2007 [49]	NR	De-qi response manual	Point through point, manipulated every 6 min	25 min	NR	Two sessions Once every other day	NR

Hou et al. 2009 [50]	NR	NR	EA	20 min	NR	Once a day for three weekdays	NR

Wen et al. 2005 [51]	13–40 mm	De-qi response manual	manipulated every 10 min	30 min	0.35 × 40 mm	Once per day for 10 days	NR

Mu et al. 2008 [53]	NR	NR	EA	20 min	NR	Once every other day for 10 weeks (30 times)	NR

Rong et al. 2006 [55]	15–65 mm	De-qi response manual	EA	20 min	0.25 × 25 mm	Once daily for ten days	Physician

Yang et al. 2011 [54]	NR	Tolerance	EA	30 min	0.25 × 50 mm	Once per day for 12 days	NR

Zhan [52]	10–30 mm	De-qi response manual	Manipulated every 10 min	30 min	33 mm	Six times one	NR

NR: not reported, TEAS: transcutaneous electric acupoint stimulation, EA: electroacupuncture, HANS: Han's acupoint nerve stimulator, STRICTA: standards for reporting interventions in controlled trials of acupuncture.

**Table 3:** Summary of main acupoints/sites selected in the reviewed studies.

Acupoints/sites	Frequency of appearance (*N*)	Percentage (*N*/26 × %)	Acupoints appearing in the literature
Neiguan (PC6)	12	11.11	52, 47, 48, 50, 51, 45, 44, 43, 41, 39, 49, and 53
Zusanli (ST36)	10	9.26	52, 47, 48, 50, 51, 45, 44, 43, 41, and 39
Sanyinjiao (SP6)	8	7.41	52, 47, 50, 51, 45, 43, 41, and 39
Shenmen (HT7)	7	6.48	52, 47, 50, 51, 44, 43, and 53
Hegu (LI4)	6	5.56	52, 50, 45, 41, 39, and 49
Shenmen (ear)	4	3.70	38, 40, 50, and 37
Kidney (ear)	4	3.70	38, 40, 50, and 37
Liver (ear)	4	3.70	38, 40, 50, and 37
Lung (ear)	4	3.70	38, 40, 50, and 37
Sympathetic (ear)	4	3.70	38, 40, 50, and 37
Laogong (PC8)	4	3.70	50, 41, 39, and 53
Sishencong (EX-HN1)	3	2.78	45, 39, and 53
Jiaji (EX-B2)	3	1.85	43, 51, and 47
Shenshu	3	1.85	47, 51, and 43
Taichong	2	1.85	47 and 48
Baihui (GV20/DU20)	2	1.85	44 and 42
Waiguan (SJ5)	2	1.85	52 and 41
Zhiyang (GV9)	2	1.85	48 and 42
Dazhui (GV14/DU14)	1	0.93	42
Mingmen (GV4)	1	1.85	42
Shendao (GV11)	1	0.93	42
Lingtai (GV10)	1	0.93	42
Shenting	1	0.93	53
Naokong	1	0.93	53
Yintang	1	0.93	53
Yangbai	1	0.93	53
Yongquan	1	0.93	53
Quanzhong	1	0.93	53
Naohu	1	0.93	53
Ben Shen	1	0.93	53
Fengchi	1	0.93	48
Anmian	1	0.93	48
